# Correspondence

**Published:** 1908-02

**Authors:** J. M. Prichard

**Affiliations:** Olinger, Va.


### CORRESPONDENCE

Olinger, Va., Nov. 19th 1907.

Editor Journal-Record of Medicine:—

You are right in supposing that every doctor with a record of thirty-six years practice has had cases worth relating, and that he has numbers of—to him—sure remedies in certain given conditions; that he has some very useful methods—from experience—methods which he regards as almost sacred, and a profouud secret known only to himself. All of these should be published, criticized, proved and adopted, or disproved and discarded. The journal which can get the innermost secrets and thoughts from the active, working man—(not the loud mouthed) of the profession—will be a winner. Think of the history of any old tried remedy—a proved, true remedy; it generally runs back to some old woman, or like Simm's, to a negro, or to the Indians. I have a number of articles in manuscript—which I've not had time-yet, to put in good shape.

I think you are taking the right "tack" to make your Journal a great success.

Respectfully,

J. M. Prichard, M. D.

---

The committee of the Merchant's Association of New York on Pollution, has presented a lengthy report showing that the present method of disposing of the sewage of that city is not only tending to rapidly fill up the waterways and impair their usefulness for the purpose of navigation, but is also a most active agent in the spread of disease, especially typhoid fever and other intestinal disorders, which have been conclusively shown to be disseminated by house flies.

---

The state board of examiners of nurses for Georgia met January 3, 1908 at the Atlanta College of Physicians and Surgeons, for the purpose of considering applications for registration, under the law recently passed, to regulate professional nursing in the state. Sixty-six applicants were accepted.

